# Occlusive radiation cerebral vasculopathy implies medical complexity: a case report

**DOI:** 10.1186/s13256-019-2104-x

**Published:** 2019-06-03

**Authors:** Dana Ghazaleh, Azizullah Beran, Brent Berry, Malik Ghannam

**Affiliations:** 1 An-Najah National University, Nablus, Palestine; 2 Department of Gastroenterology and Hepatology, Mayo clinic, Rochester, MN USA; 3 Department of Neurology, University of Minnesota, Minneapolis, MN USA

**Keywords:** Occlusive radiation vasculopathy (ORV), Stroke, Accelerated atherosclerosis, Moyamoya, Pathogenesis, Treatment options, Computed tomographic perfusion with acetazolamide, Meningioma coexistence, Literature review

## Abstract

**Background:**

Cranial irradiation is one of the main treatment modalities for central nervous system tumors. It carries many complications, one being occlusive radiation vasculopathy of large vessels. It is an underrecognized etiology for stroke, especially in the younger population. The pathophysiological process is controversial, but there is much literature supporting the theory of its being a secondary form of moyamoya disease.

**Case presentation:**

A 31-year-old Caucasian man with a history of pineal blastoma at the age of 3 years, which was treated with resection, radiotherapy, and chemotherapy, presented to our institution with right M1 stroke. Further assessment by computed tomographic perfusion study with acetazolamide demonstrated steal phenomenon of the right middle cerebral artery territory (type III response) with a small internal region of matched cerebral blood volume defect (that is, infarct core). Coincidentally, he was found to have multiple brain masses consistent with meningiomas. Occlusive radiation vasculopathy was the most likely culprit of the patient’s stroke. The patient was treated medically with “baby” acetylsalicylic acid and clopidogrel for 3 months, then continued only on baby acetylsalicylic acid.

**Conclusion:**

Late-onset occlusive radiation vasculopathy is a potentially severe iatrogenic manifestation of radiotherapy that requires a high index of suspicion as an etiology of stroke in young population, especially those with coexistent meningioma that might be a strong indicator for occlusive radiation vasculopathy as the stroke culprit. We reviewed the available literature to better understand the pathogenesis, clinical presentation, and treatment options of occlusive radiation vasculopathy. Applying perfusion studies with acetazolamide measures the cerebrovascular reserve in patients with occlusive radiation vasculopathy, which could help in determining the appropriate available treatment option.

## Background

Cranial irradiation is one of the main treatment modalities for central nervous system tumors. In the past, patients with cancer usually did not live long enough to experience many long-term complications of radiation, because they often died of the malignancy itself [1]. Nowadays, cancer survivors are growing in number owing to improvement in the quality of care. Head and neck radiation can cause substantial injury to any part of the affected region; these complications conventionally been classified temporally into acute, early delayed, and late forms. Vasculopathy of large arteries is one of the most prominent late complications. The latency time from radiation to the discovery of vasculopathy ranges broadly from 2 to 25 years [2].

Small vessels and capillaries are deemed more vulnerable than large vessels to radiation, but unlike large vessels, radiation vasculopathy affecting small vessels and capillaries occurs rather acutely, and it is much better recognized by oncologists and radiologists. This could be explained by the very nature of small vessels, having abundant endothelial cells, which are by default extremely sensitive to radiation [1]. In this article, we discuss occlusive radiation vasculopathy (ORV) as we think it is an underrecognized etiology of cerebrovascular accidents among tumor survivors, especially among young patients such as the one we describe in this report.

## Case presentation

Our patient was a 31-year-old Caucasian man with a medical history significant for pineal blastoma at the age of 3 years who had undergone tumor resection, chemotherapy, radiation, and ventriculoperitoneal shunt at that time. He presented to our institution with slurred speech, left-sided weakness, and left facial droop for the last 3 days prior to admission. His neurological examination was significant for lower left facial droop, mild dysarthria, 1/5 left lower and upper extremity strength, and some component of left-sided neglect. The patient was not given tissue plasminogen activator (tPA), because his symptoms presented outside the time window for tPA infusion.

**Fig. 1:**
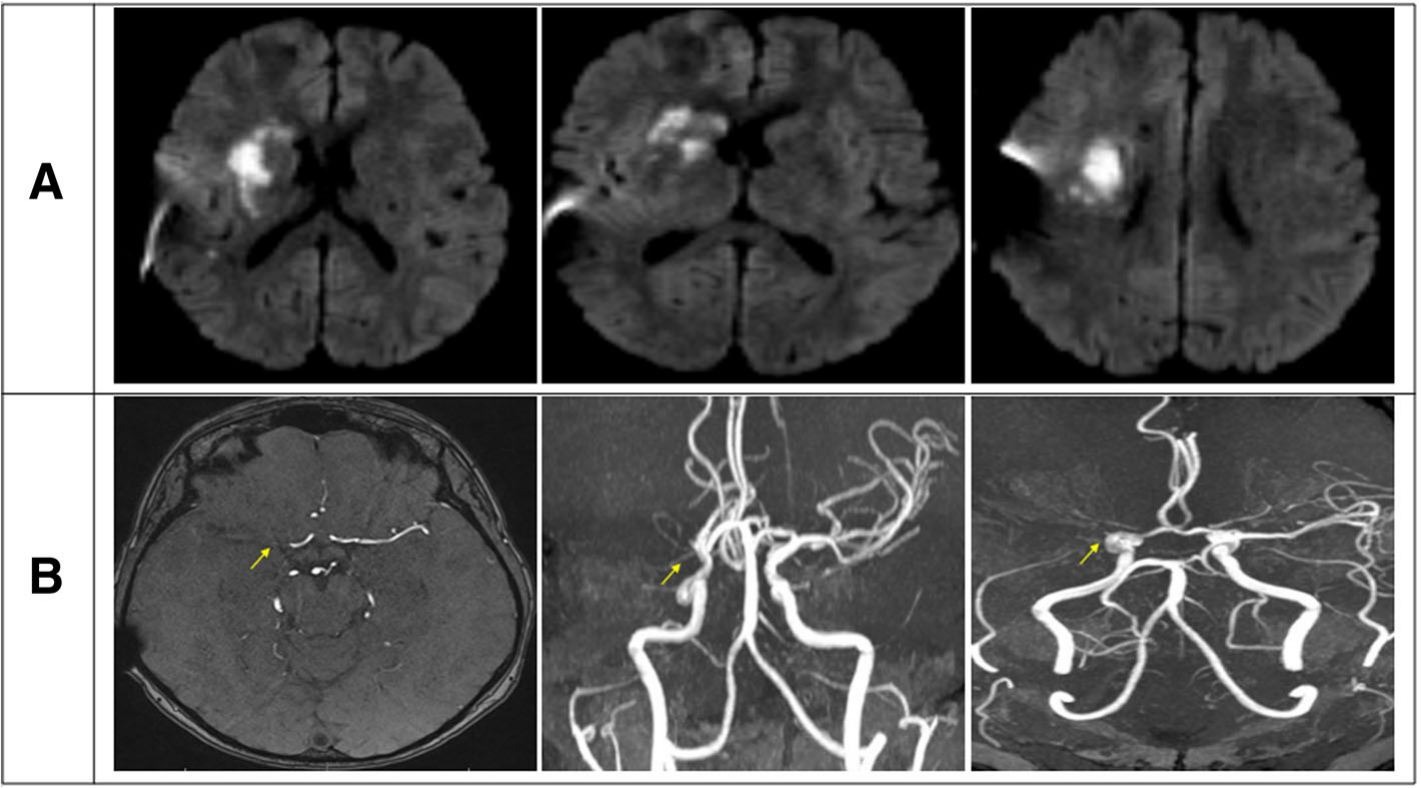
**a** Diffusion-weighted magnetic resonance imaging of the brain showing acute infarct centered in the right basal ganglia, internal capsule, external capsule, and corona radiata. **b** Magnetic resonance angiogram of circle of Willis showing complete occlusion of the right middle cerebral artery origin (*yellow arrows*)

**Fig. 2:**
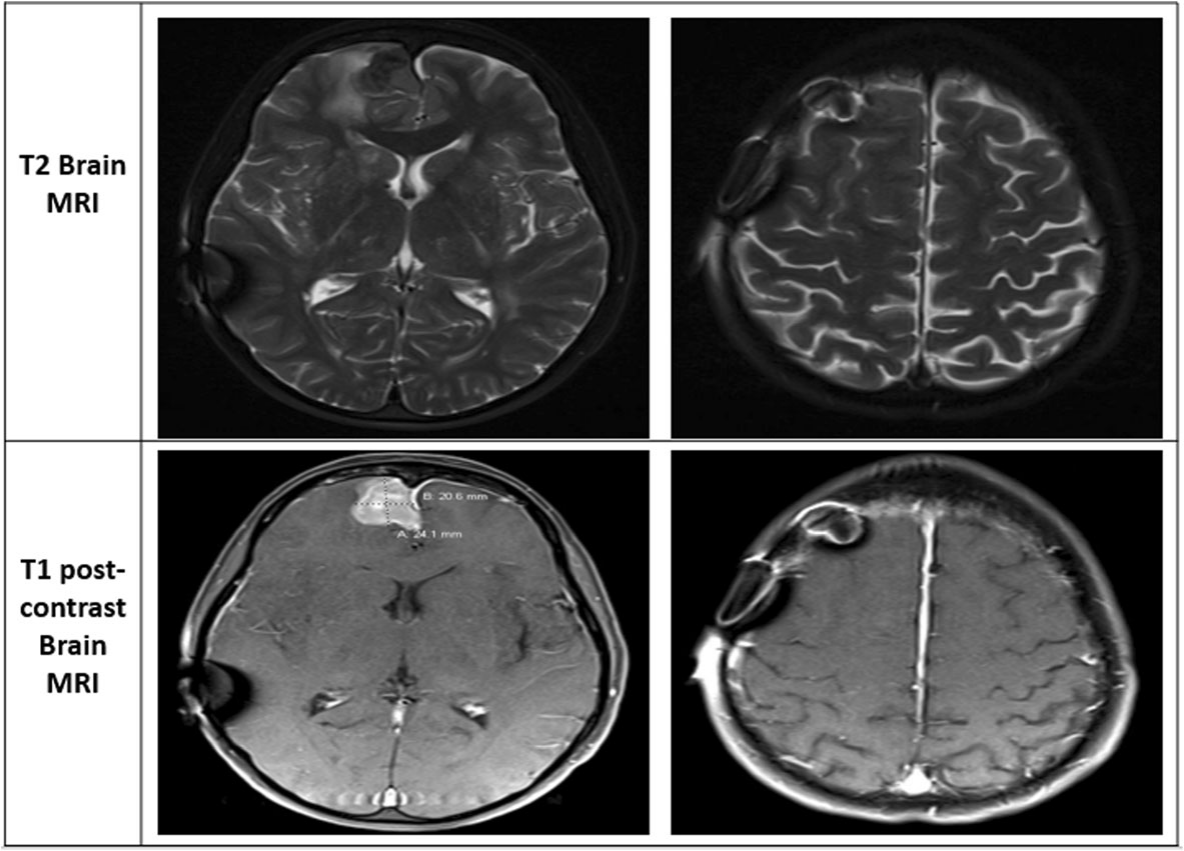
Magnetic resonance images (MRI) showing multiple enhancing intracranial lesions and extradural enhancing lesions consistent with radiation-induced meningiomas

He was found to have acute ischemic infarct of the right basal ganglia based on brain magnetic resonance imaging (MRI) (Fig. 1), as well as incidental brain masses consistent with the diagnosis of meningioma (Fig. 2). Further workup revealed right M1 occlusion on a brain magnetic resonance angiogram (Fig. 1). He was admitted for a full stroke workup that was remarkable for low-density lipoprotein of 117 mg/dl, A1C of 5.9%, uneventful echocardiogram with ejection fraction of 60–65%, no patent foramen oval, and normal atrial size. Noticeably, the patient had a hypercoagulable workup that was unremarkable. He was started on “baby” acetylsalicylic acid (ASA) 81 mg, and his atorvastatin dose was increased from 20 mg prior to admission into 40 mg.

**Fig. 3:**
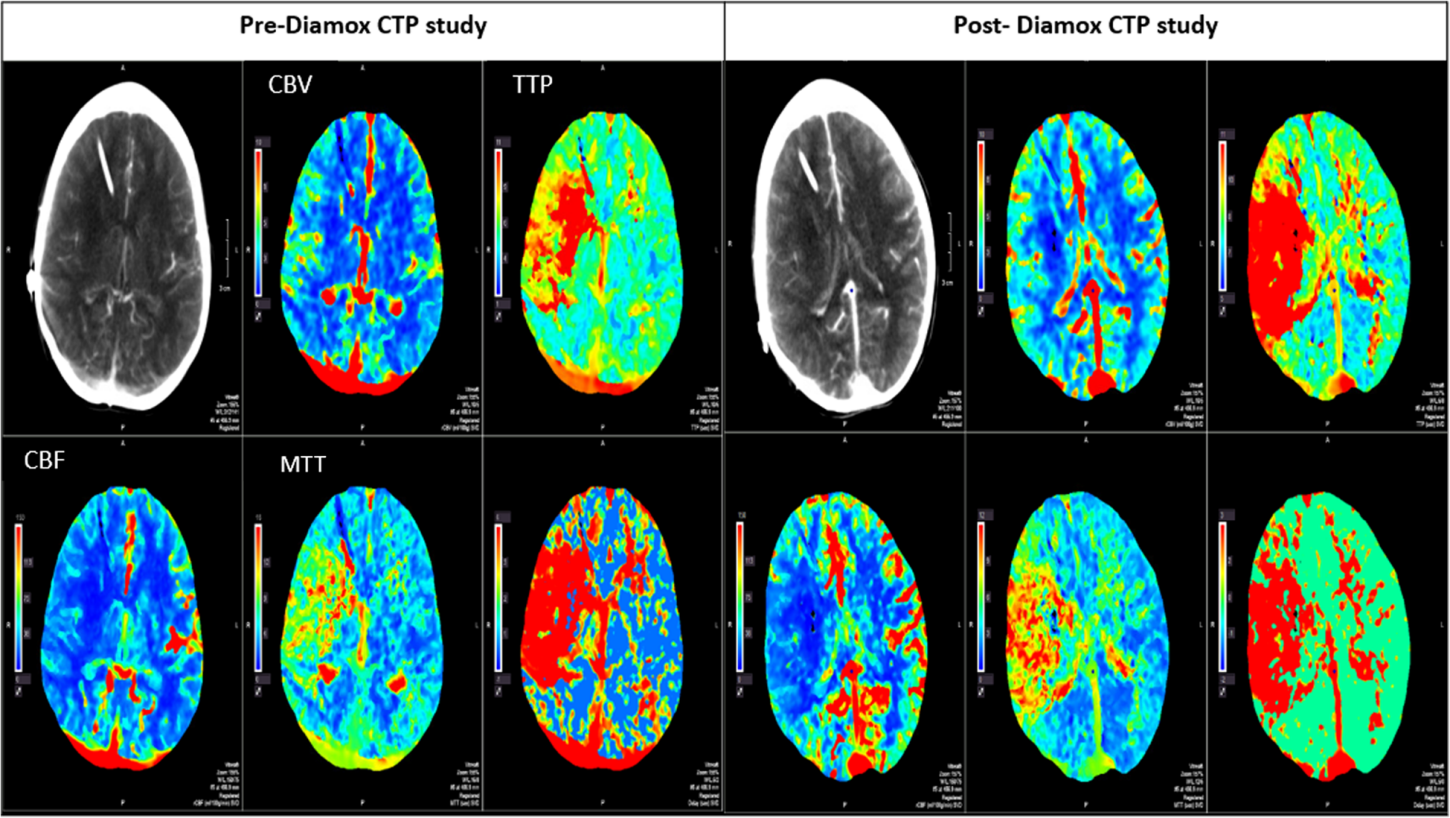
Pre-Diamox computed tomographic perfusion (CTP) study showing a large region of asymmetric diminished cerebral blood flow (CBF) and elevated mean transit time and time to peak within the right middle cerebral artery territory, with few small interspersed regions of decreased cerebral blood volume (CBV). Post-Diamox CTP study showing increased degree of asymmetric decreased CBF and elevated mean transit time (MTT)/time to peak (TTP) abnormality within the portion of the right middle cerebral artery territory, with unchanged region of matched CBV defect

**Fig. 4:**
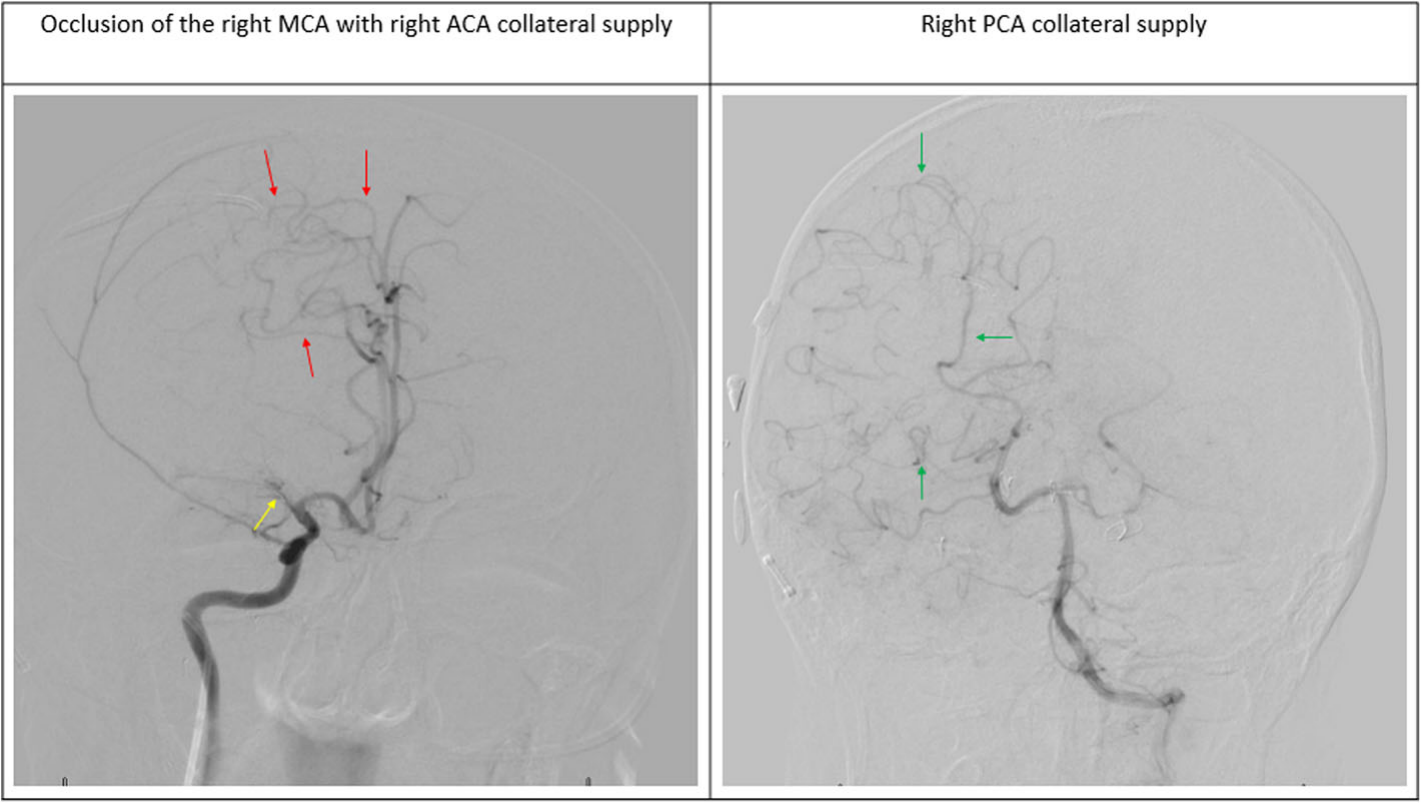
Digital subtraction angiography showing occlusion of the right middle cerebral artery (MCA) immediately after its origin from internal carotid artery (*yellow arrow*). There is collateral supply through the right MCA territory from the right anterior cerebral artery (ACA; *red arrows*) and right posterior cerebral artery (PCA; *green arrows*)

Two days after admission, the patient’s condition worsened with decreased left upper extremity and lower extremity strength. Subsequently, clopidogrel 300 mg was loaded, then the patient started on clopidogrel 75 mg daily in addition to ASA 81 mg. Repeated computed tomography of the head (CTH) and brain MRI were both stable with no worsening infarct or newly developed hemorrhage. During further investigation of the brain ischemic area, a computed tomographic perfusion (CTP) study with acetazolamide (Diamox; Teva Pharmaceuticals, North Wales, PA, USA) demonstrated evidence of baseline oligemia with post-Diamox steal phenomenon involving a large portion of the right MCA territory (type III response) with a small internal region of matched cerebral blood volume (CBV) defect (that is, infarct core). Also, the study showed evidence of pre-Diamox penumbra volume 132.52 ml and pre-Diamox infarct volume 14.97 ml (Fig. 3). A conventional cerebral angiogram showed evidence of right M1 occlusion with collateral supply from the right anterior cerebral artery and right posterior cerebral artery (Fig. 4). The neurosurgery preferred not to do bypass surgery and preferred to continue medical treatment.

After 5 admission days, the patient was discharged to the acute rehabilitation unit while receiving ASA and clopidogrel for 3 months, then only ASA for the rest of his life, in addition to atorvastatin 40 mg. He was also discharged on 30 days of cardiac event monitoring, which did not show any abnormal rhythm or atrial fibrillation later on. Three months after discharge, the patient was able to lift his left arm and leg antigravity but with spasticity that significantly improved with baclofen and botulinum toxin injection that were prescribed during his rehabilitation stay. Regarding the patient’s brain meningiomas, he was evaluated by the neurosurgery that recommended one year brain MRI follow up.

## Discussion

This report describes a 31-year-old man with a history of pineal blastoma at the age of 3 years that had been treated with resection, radiotherapy, and chemotherapy. His current presentation was right-sided M1 stroke. Further assessment by CTP with acetazolamide demonstrated steal phenomenon of the right MCA territory (type III response) with a small internal region of matched CBV defect (that is, infarct core). Coincidentally, he was found to have multiple brain masses consistent with meningiomas. ORV was the most likely cause of the patient’s stroke. The patient was treated medically with ASA and clopidogrel.

Radiation-induced cerebral vasculopathy is a wide umbrella term under which comes arterial occlusion, cerebral hemorrhage, aneurysmal formation, and cavernomas [3]. We are merely interested in the occlusive type because it was the presumptive diagnosis in our patient. ORV is an underrecognized etiology of cerebrovascular accidents among tumor survivors, and this is a challenging diagnosis to make, so a thorough history is critical to rule out any conditions that may predispose to vasculopathy, namely atherosclerosis, vasculitis, and collagen vascular disorders [4].

Many studies are in agreement on the histologic picture of the diseased blood vessels found in ORV based on autopsy findings. Almost always, there was a collection of foam cells in the intima associated with myointimal proliferation and dense hyalinization. Another major pathology noted was fibrous thickening of the tunica elastica and adventitia. All of these entities are responsible for the extreme luminal narrowing of those blood vessels [1, 5–7]. Moreover, in theory, a deterioration in prostaglandin synthesis may contribute to the whole process [8].

Radiation may take many forms: therapeutic, occupational, and environmental [9]. In this article, we focussed on the former (that is, therapeutic). The population who is receiving head and neck radiation is growing, as the therapy is not only limited to treat brain tumors, but also there is an  expanding trend of using radiation to treat cranial vascular malformations [10]. According to our litrature review, most of the reported patients of ORV had a brain tumor in childhood or adolescence. This age association is mostly attributed to the fact that the developing vascular system in the younger population is vulnerable to harmful stimuli, including radiation [6].

Many cancer types have been documented to give rise to vasculopathy after radiation therapy (Table 1), but there seems to be a predilection in tumors arising from the optic pathway or anything close to the sella turcica [6], probably due to the proximity of this area to the major intracerebral blood vessels [21]. One study clearly showed that radiation therapy for optic pathway glioma was associated with a relative risk of 4.1 for development of cerebrovascular accident (CVA) [22]. Nevertheless, no part of the brain seems to be exempt from this phenomenon.

**Table 1:** Reported cases of radiation-induced intracranial vasculopathy

Reference	Age (years)	Cancer type	Latency to the documented vascular lesion (year/s)
[7]	57	Fourth ventricular ependymoma	0.3
[7]	26	Right insular oligodendroglioma	10
[11]	10	Craniopharyngioma	4
[11]	2.5	Atypical teratoid rhabdoid tumor	1
[11]	15	Medulloblastoma	8
[11]	9	Craniopharyngioma	1
[11]	5	Anaplastic astrocytoma	2
[1]	34	Right thalamic astrocytoma	18
[1]	46	Anaplastic astrocytoma	9
[12]	9	Optic chiasmatic glioma	9
[12]	17	Optic nerve glioma	2
[10]	31	Craniopharyngioma	19
[10]	15	Germinoma	5
[10]	8	Optic glioma	7
[13]	68	Pituitary adenoma	8
[14]	20	Pituitary adenoma	7
[15]	0.5	Optic nerve astrocytoma	14.5
[16]	12	Craniopharyngioma	2
[16]	34	Suprasellar germinoma	9
[16]	3	Pilocytic astrocytoma	2
[17]	8	Optic glioma	4
[17]	7	Retinoblastoma	5.5
[17]	29	Medulloblastoma	23
[18]	20	Hodgkin’s disease	5
[19]	6	Optic glioma	3
[8]	38	Anaplastic astrocytoma	12
[6]	19	Craniopharyngioma	12
[20]	3	Hypothalamic tumor	0.5
[20]	11	Periventricular astrocytoma	2.5

Different types of radiation have been associated with vasculopathy, with some studies comparing the consequence of proton beam therapy with the conventional fractionated photon therapy, but no statistical difference was found [11, 23]. Although the dose effect is unknown, some studies claim that the effect is largely dependent on the dose and volume of radiation provided, which is associated with earlier presentation if the dose and or volume is higher [24, 25]. It is still unknown what makes some individuals more susceptible than others to ORV. However, identifying the predictors for developing ORV would aid in the process of prevention and would allow for more cautious monitoring [23]. There are possible genetic factors that could help clinicians predict part of that association, and these are currently under investigation [26].

Besides case studies and series, other large-scale studies have been conducted to estimate the risk of radiation vasculopathy. Arthurs *et al.* compared the effect of radiation therapy with that of surgery in the development of stroke. Their results significantly showed that patients treated with radiation alone had a hazard ratio of stroke that was 1.70 times higher than the hazard ratio for surgery alone (*P* < 0.001) [27]. The exact incidence of ORV is largely variable with many determinants, including tumor type and location, radiation dose, and follow-up time. A study conducted in Taiwan that included 391 children who had brain tumors with a 7-year follow-up showed the crude incidence of postradiotherapy moyamoya syndrome to be 33.3% for optic glioma, 12.5% for craniopharyngioma, 2% for medulloblastoma, and 5.3% for astrocytoma [28]. These incidence rates were close to those reported in two other series, one from the United States [29] and the other from Korea [30].

Because no definitive histopathologic study exists apart from animal models and some case series, the pathogenesis of ORV is still not quite clear [4]. Some have postulated that ORV is a consequence of accelerated atherosclerosis [2, 27]. Others have claimed it to be moyamoya syndrome [6, 11]. Moyamoya disease is a relatively rare cerebrovascular disorder characterized by progressive occlusion of the medium and large cerebral arteries in the circle of Willis [31]. This occlusion triggers the formation of a fine collateral network of vessels at the base of the brain, giving the appearance of cigarette smoke on an arterial angiogram, hence the name “moyamoya,” which is the Japanese translation of the term “puff of smoke” given in 1969 by Suzuki and Takaku [5]. This vasculopathy traditionally occurs with no apparent cause or associated neurological disorder in the so-called “primary or true moyamoya disease.” However, a new term has been introduced: “moyamoya syndrome,” which comprises basically the same vasculopathic changes but occurs due to secondary underlying causes such as neurofibromatosis; arteriosclerosis; Down syndrome; sickle cell disease; or, as in our patient, a manifestation of radiation therapy [6]. In this article, we chose to use the description “occlusive radiation vasculopathy” instead of moyamoya because the majority of cases [11], including our patient’s, do not show angiographically evident collateral vessels that are seen in moyamoya. However, having minimal collateral vessels could mean that the process was not chronic enough, making early identification by imaging techniques more challenging.

Management of stroke secondary to ORV will always be challenging as long as no clear guidelines exist on how to approach similar patients. A review of the literature reveals that management for ORV is still vague and ill-defined [4]. The Stenting vs. Aggressive Medical Management for Preventing Recurrent Stroke in Intracranial Stenosis (SAMMPRIS) trial, which focused on atherosclerotic intracranial arterial stenosis, showed that aggressive medical management was superior to stenting for secondary stroke prevention, but it did not mention radiation-induced vasculopathy [32]. Even though this does not look like atherosclerosis, especially in patients of this young age, it was essential to exclude atherosclerosis and other diseases from the picture. Many factors made atherosclerosis unlikely in our patient, including the absence of significant risk factors such as hypertension, hyperlipidemia, and smoking. Also, having a minimal amount of atherosclerosis in the extracranial and nonirradiated arteries supported this exclusion. Erythrocyte sedimentation rate and antineutrophil cytoplasmic antibody tests were ordered to take vasculitis out of the picture, and the results were negative. The patient’s family history was negative regarding any hereditary diseases with similar manifestations. It is still controversial whether treatment should mimic that used for other atherosclerotic strokes, because the pathophysiology is not agreed upon. Medical management itself is still not adequately assessed as an effective treatment option, but many support the use of antiplatelets and anticoagulants. It is also unknown if risk factor modification with antihypertensives and lipid-lowering agents plays a role in secondary stroke prevention in ORV cases [33].

On the other hand, strategies that can enhance collateral perfusion or alleviate occlusions have started to gain popularity [33]. Surgical bypass procedures have been reported to yield positive outcomes in a few small series, but there are no reliable data on the basis of which to give recommendations [34]. Surgery may carry many complications in patients with a previous history of radiation [25], so alternative options such as carotid angioplasty and stenting could replace endarterectomy [2]. CTP with acetazolamide (Diamox) is commonly used to assess the collateral vessels before deciding on any intervention. Patients who have chronic steno-occlusive disease try to compensate by autoregulatory vasodilation in an attempt to maintain cerebral blood flow (CBF). The presence of this vasodilatory capacity reflects what is known as cerebrovascular reserve (CVR). The flow reserve can be assessed best by perfusion studies such as CTP, using acetazolamide (Diamox) as a challenge agent to induce vasodilation [35, 36]. Many parameters are measured before and after the administration of Diamox, most importantly CBF, CBV, and mean transit time. Rogg *et al.* classified patients’ responses to Diamox into three types. Type I patients have normal baseline CBF that increases after Diamox administration. Type II patients have areas of decreased baseline CBF that increase after Diamox challenge. Type III patients have decreased CBF at baseline with a paradoxical decrease in blood flow after Diamox, likely due to shunting of blood away from the underperfused region (steal phenomenon) [37]. This challenge test is a useful clinical tool to estimate disease severity and future ischemic risk. It also aids in selecting the appropriate therapeutic intervention [35].

The development of secondary tumor after radiation is a complication with one of the highest fatality rates in the treatment of cranial malignancies. Several tumor types have been reported. Meningioma has been shown to be the most common radiation-induced tumor in adults and the second most common in children [38]. These meningiomas seem to be distinct from primary meningioma in being more aggressive, with a higher rate of multiplicity and recurrence [3]. The coexistence of radiation-induced tumor and occlusive vasculopathy has rarely been reported. In their report of two cases in 1985, Montanera *et al.* were the first to document the coexistence of these entities [39]. Another case reported later was anaplastic meningioma found after 19 years of radiation therapy, in which angiography was performed and showed large vessel vasculopathy [40]. The third study of such coexistence was in a patient with medulloblastoma [3]. In our patient, three meningiomas were found incidentally at the time of presentation for CVA, and, given the fact of remote cranial radiation therapy with meningioma coexistence, ORV was the most suggesed etiology of our patient's stroke.

Apart from this unique coexistence, the purpose of this article is also to give an update on ORV by shedding light on the key studies that helped us to better understand the pathogenesis, clinical presentation, and treatment of the disease. For future research, we hope there would be more focus on medical and surgical management options for ORV, especially for secondary stroke prevention, with a well-established approach to provide timely evidence-based care for such patients.

## Conclusion

Late-onset ORV is a potentially severe iatrogenic manifestation of radiotherapy that requires a high index of suspicion as an etiology of stroke in young population, especially in those patients with meningioma coexistence that might be a strong indicator for ORV as the stroke culprit. We reviewed the available literature to better understand the pathogenesis, clinical presentation, and treatment options of ORV. Applying perfusion studies with acetazolamide can measure the CVR in patients with ORV, which could help in determining the appropriate available treatment option.

## Abbreviations

ACA: Anterior cerebral artery
ASA: Acetylsalicylic acid
CBF: Cerebral blood flow
CBV: Cerebral blood volume
CTP: Computed tomographic perfusion
CVA: Cerebrovascular accident
CVR: Cerebrovascular reserve
MCA: Middle cerebral artery
MRI: Magnetic resonance imaging
MTT: Mean transit time
ORV: Occlusive radiation vasculopathy
PCA: Posterior cerebral artery
SAMMPRIS: Stenting vs. Aggressive Medical Management for Preventing Recurrent Stroke in Intracranial Stenosis trial
tPA: Tissue plasminogen activator
